# Validation of an in-house HPV CerviSens self-sampling kit: comparison against clinician-collected and self-collected samples

**DOI:** 10.3389/fmedt.2025.1458857

**Published:** 2025-09-19

**Authors:** Preeti Arora, Shruti Jawale, Sanjay Gupte, Sarjan Shah

**Affiliations:** ^1^ Greenarray Genomic Research and Solutions Pvt. Ltd., Kothrud, Pune, India; ^2^ Gupte Hospital and Research Centre, Kothrud, Pune, India

**Keywords:** self-sampling kit, human papillomavirus, CA cervix, cervical cancer, high-risk HPV, self-sampling, HPV screening

## Abstract

**Introduction:**

Cervical cancer (CA cervix) ranks as the second most common cancer among women aged 15–44 and remains a leading cause of cancer-related mortality. Regular screening for cervical cancer significantly reduces mortality rates. Due to the strong causal relationship between high-risk human papillomavirus (hrHPV) and cervical cancer, HPV DNA testing has been developed as a screening method. HPV self-sampling kits have the potential to increase screening uptake, facilitate early detection, and reduce the global burden of cervical cancer. This study evaluates the efficacy of an in-house developed HPV CerviSens self-sampling kit for women in detecting hrHPV types.

**Methodology:**

The study, approved by the Gupte Hospital Ethics Committee, included women aged 35–65 visiting Gupte Hospital in Pune, India. Participants self-collected vaginal samples using the in-house developed HPV CerviSens kit, and trained healthcare practitioners collected conventional samples. HPV DNA analysis was performed using the Cobas 4800 assay. Concordance between self-sampling and clinician sampling was assessed using Cohen's *κ* statistic. The sensitivity and specificity of HPV detection in self-samples were calculated with clinician-collected samples as the reference standard.

**Results:**

A total of 203 paired self-collected and clinician-collected samples were analyzed for HPV detection. The median age of participants was 44 years. Concordance for HPV detection between self-samples and clinician-collected samples was very good (Cohen's *κ*: 0.88, 95% CI: *κ* ≥ 0.81). For HPV detection in self-samples, the in-house HPV CerviSens self-sampling kit demonstrated a sensitivity of 98.0% (95% CI: 89.4%–99.9%) and a specificity of 99.4% (95% CI: 96.3%–99.9%) when clinician-collected samples were used as the reference standard. These results demonstrate that the self-sampling method provides high accuracy in identifying high-risk HPV infections.

**Conclusion:**

HPV self-sampling using the in-house developed HPV CerviSens kit is a reliable and effective method for cervical cancer screening, with high concordance and accuracy in detecting HPV infections. Integrating self-sampling into screening programs can enhance early detection, improve patient outcomes, and significantly reduce the global burden of cervical cancer.

## 1 Introduction

Cervical cancer (CA cervix) ranks as the second most common cancer among women aged between 15 and 44 years (1). It is a leading cause of cancer-related mortality, accounting for 17% of all cancer deaths among women. However, CA cancer screening has been shown to significantly reduce mortality (2). Unlike many other cancer types, CA cervix is amenable to screening for early diagnosis and intervention. Conventional cervical cancer screening methods, such as Pap smears, liquid-based cytology (LBC), human papillomavirus (HPV) testing, VIA/VILI, and colposcopy, have limitations compared with self-sample collection devices for HPV screening. These methods often require access to healthcare facilities and trained personnel, posing barriers in rural or underserved areas. They can also be costly due to provider fees and sample processing, whereas self-sample devices are cost-effective, allowing at-home testing without travel expenses. Unlike traditional approaches such as Pap smears or LBC, which require clinical facilities and trained personnel for sample collection, self-sampling devices allow women to collect cervical samples conveniently at home. HPV self-testing represents a significant breakthrough in cervical cancer screening, addressing several limitations of conventional methods. Self-testing reduces costs associated with clinic visits and improves privacy and comfort, potentially increasing screening compliance rates. Studies have shown that self-collected samples for HPV testing are comparable in quality to clinician-collected samples, ensuring reliable results. By empowering women to take charge of their health through easy and private sample collection, HPV self-testing enhances early detection efforts and contributes to reducing the burden of cervical cancer. Integrating self-sampling into screening programs can effectively complement existing methods, broaden access, and improve overall screening effectiveness across diverse populations.

In addition, HPV self-sampling kits have the potential to increase screening uptake, facilitate early detection, and therefore reduce the global burden of cervical cancer. Furthermore, HPV-based screening is more sensitive than cytology-based screening for detecting cervical intraepithelial neoplasia grade 3 (CIN3) and cervical cancer (3, 4).

A systematic review has shown that self-sampling is a well-accepted method for cervical cancer screening (5). One advantage of HPV testing is that it allows women to self-sample cervico-vaginal material at home (HPV self-sampling), which may enhance participation in cervical cancer screening programs (6). Comparative studies between self-sampled and clinician-collected samples for HPV detection have shown moderate to very good concordance in referral populations (7, 8), and one study in a screening population reported a very high level of agreement (9). Cost–consequence analyses also suggest that self-sampling strategies may offer a less expensive alternative for HPV primary screening, potentially saving significant healthcare costs while expanding screening accessibility to under-screened populations. This study aims to evaluate the efficacy of the HPV CerviSens self-sampling kit for women in detecting human papillomavirus (HPV) types and predicting cervical lesions. We studied the concordance in identifying high-risk HPV (hrHPV) types between clinician-collected samples and those self-collected by women using the in-house developed HPV CerviSens self-sampling kit for validation and use in clinical settings.

## 2 Methodology

### 2.1 Study center and sample acquisition

The present study was approved by the Institutional Ethics Committee (Certificate No. GHEC/2022-23/013). All research involving human subjects was conducted in accordance with the principles of the Declaration of Helsinki. Women visiting Gupte Hospital in Pune, India, were included in the study. The inclusion and exclusion criteria are listed below.

#### 2.1.1 Inclusion criteria

Women aged between 35 and 65 years.

#### 2.1.2 Exclusion criteria

• Pregnancy and within the first 6 weeks of the puerperium
• Absence of a cervix (due to prior hysterectomy or trachelectomy)
• Vaginal bleeding
• Use of medications, creams, or vaginal douches in the last 48 h before sample collection
• Inability to perform self-sampling

**Figure 1:**
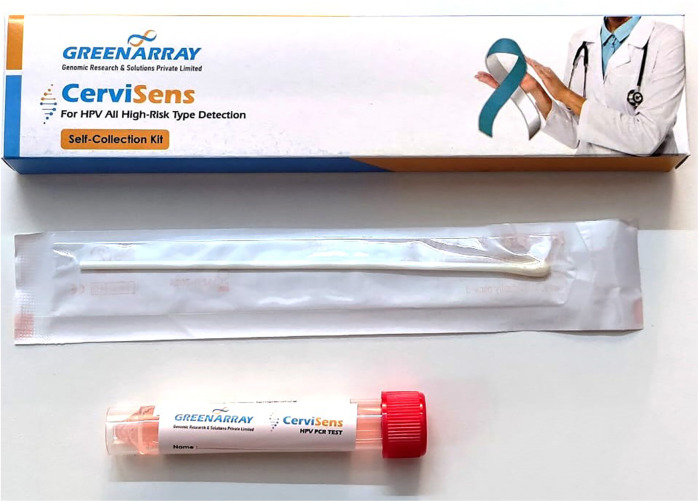
HPV CerviSens kit components.

Women provided two vaginal samples for HPV testing—one self-collected using the in-house developed HPV CerviSens self-sampling kit and another collected by trained healthcare practitioners at the hospital. Prior to sample collection, participants received verbal and written instructions with illustrations and gave informed consent to participate in the study. The in-house developed HPV CerviSens self-sampling kit for HPV detection includes a sterile screw cap tube prefilled with 2 ml of transport media, ensuring the integrity of the sample during transport (Figure 1). The kit also contains a cotton swab affixed to the end of a plastic rod with a marked breakpoint for ease of use.

**Figure 2:**
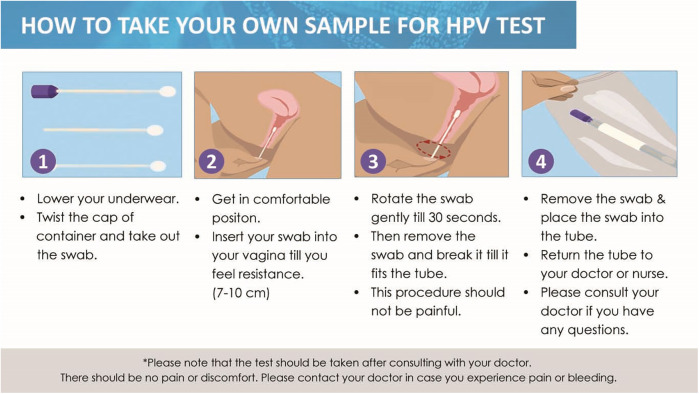
HPV CerviSens user instructions.

Detailed instructions are provided in the kit insert, guiding users on the correct procedure for swab collection, thus facilitating accurate and reliable self-sampling for cervical cancer screening (Figure 2).

The specimen was obtained and stored at 4°C. The collected samples were used for HPV infection detection and genotyping. To minimize potential bias introduced by the order and timing of sample collection, we ensured that clinician-collected samples were obtained within 1 h of self-sampling. This timing was chosen to reduce the risk of time-dependent HPV DNA degradation. Additionally, self-sampling was performed first to avoid any bleeding or disruption of the cervical environment caused by speculum use, which could affect the accuracy of the self-collected sample. This sequence helped preserve the integrity of both samples and minimized the likelihood of one sampling method influencing the other.

For the comparison of the two collection systems, only the 14 hrHPV types were evaluated, including 16, 18, 31, 33, 35, 39, 45, 51, 52, 56, 58, 59, 66, and 68. Results were considered identical when both samples contained the same set of hrHPV genotypes. Samples were classified as concordant if at least one hrHPV genotype was shared between the two samples, even if the complete genotype sets differed. Discordant results were defined when no overlapping hrHPV genotypes were detected between the paired samples. This classification allowed for a nuanced assessment of agreement between the two collection methods.

### 2.2 HPV infection detection by polymerase chain reaction

For HPV DNA analysis, the Cobas 4800 assay (Roche Diagnostics, Switzerland) was employed. This fully automated real-time PCR method separately detects HPV16, HPV18, and 12 other hrHPV types (HPV 31, 33, 35, 39, 45, 51, 52, 56, 58, 59, 66, and 68), utilizing the β-globin gene as an extraction and amplification control. The Cobas HPV test is a highly automated assay designed to detect hrHPV DNA in LBC specimens using real-time PCR technology. It utilizes a set of 16 PCR primers (8 forward and 8 reverse) to amplify a ∼200 bp fragment of the L1 gene from all 14 hrHPV genotypes. TaqMan probes, labeled with three spectrally unique fluorescent dyes, enable the simultaneous detection of these 14 hrHPV types across three separate channels using real-time PCR technology. In channel 1, 12 hrHPV types (HPV 31, 33, 35, 39, 45, 51, 52, 56, 58, 59, 66, and 68) are detected as a pool. Channel 2 specifically detects HPV16, while channel 3 detects HPV18. Channel 4 detects a 330 bp amplicon from the human β-globin gene, serving as a control for sampling adequacy; a positive β-globin result confirms the presence of human cells in the collection vial (10, 11). All samples were processed in the same way for both self-collected and clinician-collected samples. In addition, the same processing team was maintained throughout to ensure that protocols were uniformly followed for all samples.

### 2.3 Statistical analysis

The concordance of HPV detection between paired samples was evaluated using Cohen's kappa statistic (*κ*), categorized as follows: “poor” (*κ* ≤ 0.20), “fair” (0.21 ≤ *κ* ≤ 0.40), “moderate” (0.41 ≤ *κ* ≤ 0.60), “good” (0.61 ≤ *κ* ≤ 0.80), and “very good” (*κ* ≥ 0.81). The overall agreement percentage was calculated by dividing the number of concordant sample pairs by the total number of samples. The sensitivity and specificity of HPV detection in self-samples were calculated with 95% CIs using the binomial distribution, with clinician-collected samples serving as the reference standard. For specific HPV genotypes (HPV16/18 and other HPV types), genotypes were classified as “HPV16/18” (HPV16 and/or HPV18, including co-infections with other types) and “HPV other” (HPV types other than 16/18, including co-infections with HPV16/18). Concordance was defined as the presence of at least one identical genotype in both samples, while discordance was defined as the absence of genotype similarities. *P*-values <0.05 were considered statistically significant. All analyses were performed using SPSS version 17.0 for Windows (Chicago, IL, USA).

## 3 Results

**Figure 3:**
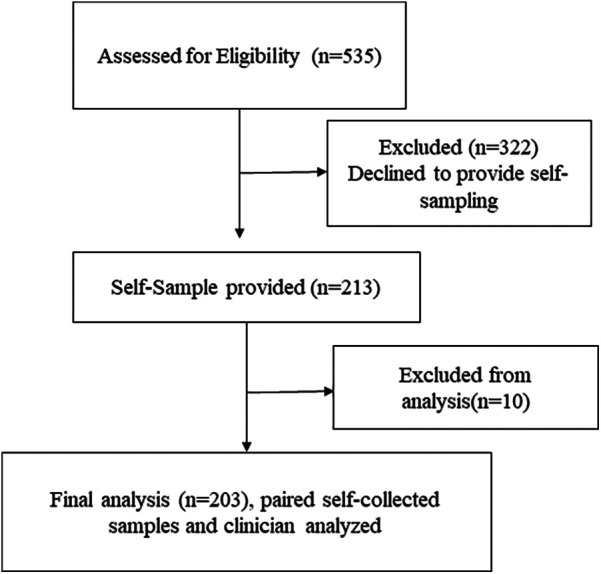
CONSORT-style flow diagram depicting participant progression through the HPV self-sampling diagnostic accuracy study.

Out of 535 eligible women, 213 (39.8%) agreed to provide a self-sample. Ten women were excluded because their self-samples were taken after the biopsy, leaving 203 women for analysis. The median age of the included women was 44 years (IQR: 38–49 years). The majority aged 40–49 years (*n* = 130, 61.0%), followed by those aged 30–39 years (*n* = 48, 22.5%), and 50–59 years (*n* = 35, 16.4%). All paired clinician-collected samples and self-samples were valid for HPV testing. Figure 3 presents a CONSORT-style flow diagram depicting participant progression through the HPV self-sampling diagnostic accuracy study.

### 3.1 Concordance between self-sampling and clinician sampling

There was a very good concordance for HPV detection between the self-samples and the clinician-collected samples (Cohen's *κ*: 0.88, 95% CI: *κ* ≥ 0.81) (Table 1). For HPV detection in self-samples, the in-house HPV CerviSens self-sampling kit demonstrated a sensitivity of 98.0% (95% CI: 89.4%–99.9%) and a specificity of 99.4% (95% CI: 96.3%–99.9%) when clinician-collected samples were used as the reference standard.

**Table 1:** Concordance for HPV detection between self-samples and clinician-collected samples.

Self-collected samples (*n*)			Clinician-collected sample (*n*)			*κ* (95% CI)	Sensitivity	Specificity
HPV positive	HPV negative	Total samples	HPV positive	HPV negative	Total			
50	153	203	49	154	203	0.88 *κ*:, 95% CI: *κ* ≥ 0.81	98.1% (95% CI: 66.7%–90.9%	99.3% (95% CI: 86.3%–95.3%)

HPV any positive: HPV16 and/or HPV18 and/or HPV31, 33, 35, 39, 45, 51, 52, 56, 58, 59, 66, and 68. Cohen's kappa: “poor” (*κ* ≤ 0.20), “fair” (0.21 ≤ *κ* ≤ 0.40), “moderate” (0.41 ≤ *κ* ≤ 0.60), “good” (0.61 ≤ *κ* ≤ 0.80), or “very good” (*κ* ≥ 0.81).

### 3.2 Concordance between self-sampling and clinician sampling according to specific genotypes

Concordance for HPV16/HPV18 detection between self-samples and GP-collected samples was very good with an overall agreement of 98% (95% CI: 92.1%–98.0%) (Table 2). For other HPV types, a good concordance was seen between self-samples and GP-collected samples (*k* = 0.91, 95% CI: 0.51–0.95), with an overall agreement of 88.7% (95% CI: 83.7%–92.6%) (Table 2).

**Table 2:** Concordance and agreement between self-samples and clinician sampling according to specific genotypes.

Self-samples (*n*)				Clinician-collected sample (*n*)				*κ* (95% CI) HPV 16/18	*κ* (95% CI) other positive
HPV 16/18 positive	HPV other positive	Total positive samples	Total negative samples	HPV 16/18 positive	HPV other positive	Total positive	Total negative samples		
43	7	50	153	43	6	49	154	0.98	0.91

Cohen's kappa: “poor” (*κ* ≤ 0.20), “fair” (0.21 ≤ *κ* ≤ 0.40), “moderate” (0.41 ≤ *κ* ≤ 0.60), “good” (0.61 ≤ *κ* ≤ 0.80), or “very good” (*κ* ≥ 0.81). HPV16/18: HPV16 and/or HPV18 including co-infections with HPV of other types (HPV31, 33, 35, 39, 45, 51, 52, 56, 58, 59, 66, and 68). HPV other: HPV31, 33, 35, 39, 45, 51, 52, 56, 58, 59, 66, and 68 including co-infections.

## 4 Discussion

Self-sampling is a feasible and acceptable approach for cervical cancer screening among women. It has been suggested that self-sampling should be considered a valuable strategy in implementing national cancer screening programs globally (12). Self-sampling for HPV screening has been extensively studied, consistently demonstrating effectiveness and potential to enhance cervical cancer screening programs. Numerous studies have explored the efficacy and benefits of self-sample collection for HPV screening. A meta-analysis by Arbyn et al., which included data from 36 studies involving a total of 154,556 women, found that self-collected samples for HPV testing are as accurate as clinician-collected samples in detecting high-risk HPV infections. These findings highlight the potential of self-sampling to improve participation in cervical cancer screening programs (13, 14). Racey et al. (15) showed that self-sampling in 818 eligible women in a small rural community in Southwestern Ontario, of whom 335 received a self-collected HPV testing kit, 331 received a reminder letter, and 152 received standard of care, increased screening rates among previously unscreened women, addressing the issue of under-screened populations. A review including 52,114 participants from studies published between 2002 and 2018, mostly cross-sectional surveys, found that self-sampling for HPV screening was more effective, feasible, and acceptable than traditional methods, suggesting its potential to expand screening coverage in underserved areas. HPV self-sampling is generally highly accepted by end users globally, making it a promising method for cervical cancer screening (16). Verdoodt et al. (6) confirmed these findings, demonstrating that self-sampling is highly acceptable among women and yields sensitivity and specificity comparable to clinician-based sampling. These studies collectively demonstrate that self-sample collection for HPV screening is a reliable, acceptable, and effective method for increasing cervical cancer screening rates.

In the present study, we have demonstrated a strong agreement in HPV detection between self-collected samples and clinician-collected samples. The HPV CerviSens self-sampling kit emerged as a highly accepted method for screening. Importantly, our findings indicate that self-sampling did not miss any cases of underlying cervical intraepithelial neoplasia grade 2 or higher (CIN2+), compared with clinician-collected sampling. This highlights the reliability and effectiveness of HPV self-sampling kits in cervical cancer screening programs. A significant strength of our study lies in the utilization of both a clinically validated self-sampling device and an automated PCR-based HPV DNA test assay on paired samples (11). Moreover, self-samples were collected by women at home without direct supervision from healthcare professionals, mirroring real-world conditions essential for evaluating the efficacy of self-sampling prior to its integration into routine screening programs.

We evaluated the concordance and performance of HPV self-sampling compared with clinician-collected samples in a cohort of 213 women undergoing cervical cancer screening. We found very good concordance for HPV detection between self-samples and clinician-collected samples, as indicated by a Cohen's kappa of 0.88 (95% CI: ≥0.81), which suggests substantial agreement. This finding supports previous research indicating that self-sampling is a reliable method for HPV detection, comparable to sampling performed by healthcare professionals (14, 17). For HPV detection in self-samples, the in-house HPV CerviSens self-sampling kit demonstrated a sensitivity of 98.0% (95% CI: 89.4%–99.9%) and a specificity of 99.4% (95% CI: 96.3%–99.9%) when clinician-collected samples were used as the reference standard. The high sensitivity and specificity for HPV detection in self-samples further validate the accuracy of self-sampling kits in identifying HPV infections. The convenience and ease of use of self-sampling significantly enhance patient compliance and accessibility. Women can collect samples in the comfort of their own homes, eliminating the need for clinical visits and associated logistical challenges. This aspect is particularly beneficial for increasing screening coverage in underserved populations and remote areas with limited access to healthcare facilities.

Our study also examined the concordance for specific HPV genotypes. We observed a very good agreement (*κ* = 0.98, 95% CI: 92.1%–98.0%) for HPV16/HPV18 detection between self-samples and clinician-collected samples, indicating robust performance in detecting these high-risk genotypes. For other HPV types, the concordance was also very good (*κ* = 0.91, 95% CI: 0.51–0.78), underscoring the kit's ability to detect a broad range of HPV infections, further validating its utility in comprehensive cervical cancer screening programs. Our study demonstrated that HPV self-sampling could serve as an effective alternative to clinician-based sampling in cervical cancer screening programs. This approach is particularly valuable for increasing screening uptake among underserved populations and reducing barriers associated with clinic visits. The findings align with global efforts to improve cervical cancer prevention and control through accessible patient-centered screening strategies, as advocated by organizations such as the World Health Organization (18, 19).

The HPV CerviSens self-sampling kit exhibited high sensitivity and specificity in detecting HPV infections. Specifically, the sensitivity of the kit was 98.1% (95% CI: 66.7%–90.9%), indicating that it accurately identified nearly all true positive cases of HPV infection, and the specificity was 99.3% (95% CI: 86.3%–95.3%), demonstrating that it effectively distinguished between HPV-positive and HPV-negative samples, with a minimal rate of false positives. These performance metrics are crucial for ensuring that the self-sampling method is reliable and can be trusted to provide accurate results comparable to those obtained through clinician-based sampling. The convenience and ease of use of the HPV CerviSens self-sampling kit significantly enhance patient compliance and accessibility. Women can collect samples in the comfort of their own homes, eliminating the need for clinical visits and associated logistical challenges. This aspect is particularly beneficial for increasing screening coverage in underserved populations and remote areas with limited access to healthcare facilities. Additionally, the self-sampling method respects the privacy and autonomy of women, which can encourage more individuals to participate in regular screening.

## 5 Conclusion

HPV self-sampling using the in-house developed HPV CerviSens kit demonstrated high concordance and accuracy in detecting HPV infections, supporting its potential role in expanding cervical cancer screening programs. The high sensitivity and specificity of the kit ensure reliable detection of high-risk HPV types, while its ease of use promotes broader screening participation. Continued research and implementation efforts are warranted to maximize the benefits of self-sampling in reducing cervical cancer incidence and mortality. By integrating self-sampling into cervical cancer screening strategies, we can enhance early detection, improve patient outcomes, and ultimately contribute to the global effort to eradicate cervical cancer. The integration of this self-sampling method into screening programs holds great promise for achieving these goals and significantly reducing the global burden of cervical cancer.

## Data Availability

The raw data supporting the conclusions of this article will be made available by the authors, without undue reservation.
